# Neural hypernetwork approach for pulmonary embolism diagnosis

**DOI:** 10.1186/s13104-015-1554-5

**Published:** 2015-10-29

**Authors:** Matteo Rucco, David Sousa-Rodrigues, Emanuela Merelli, Jeffrey H Johnson, Lorenzo Falsetti, Cinzia Nitti, Aldo Salvi

**Affiliations:** School of Science and Technology, University of Camerino, Via del Bastione, Camerino, Italy; Design Complexity Group, The Open University, Milton Keynes, UK; Internal and Subintensive Medicine of Ospedali Riuniti, Ancona, Italy

**Keywords:** TOPDRIM, Hypernetworks, *Q-*analysis, Pulmonary embolism, Topology of data, Machine learning, Artificial neural network

## Abstract

**Background:**

Hypernetworks are based on topological simplicial complexes and generalize the concept of two-body relation to many-body relation. Furthermore, Hypernetworks provide a significant generalization of network theory, enabling the integration of relational structure, logic and analytic dynamics. A pulmonary embolism is a blockage of the main artery of the lung or one of its branches, frequently fatal.

**Results:**

Our study uses data on 28 diagnostic features of 1427 people considered to be at risk of pulmonary embolism enrolled in the Department of Internal and Subintensive Medicine of an Italian National Hospital “Ospedali Riuniti di Ancona”. Patients arrived in the department after a first screening executed by the emergency room. The resulting neural hypernetwork correctly recognized 94 % of those developing pulmonary embolism. This is better than previous results obtained with other methods (statistical selection of features, partial least squares regression, topological data analysis in a metric space).

**Conclusion:**

In this work we successfully derived a new integrative approach for the analysis of partial and incomplete datasets that is based on *Q-*analysis with machine learning. The new approach, called Neural Hypernetwork, has been applied to a case study of pulmonary embolism diagnosis. The novelty of this method is that it does not use clinical parameters extracted by imaging analysis.

## Background

### Introduction

Pulmonary embolism (PE) is still a disease without a set of specific clinical variables (CV), and for this reason it is known, among the medical community, as a “no-mans-land” pathology. Medical doctors (MDs) during the diagnosis task collect CV in agreement with existing guidelines [1]. MDs use Clinical Prediction Rules (CPR), that are computed using a subset of CVs, to assign to each patient a probability of being sick. The most used CPRs in diagnosing PE are Wells and Revised Geneva scores. Nevertheless, in most cases MDs still need to perform a CT-angiography to obtain a final conclusive diagnosis.

While few papers regarding advanced methods for improving detection of pulmonary embolism (PE) have been published, most of them are based on ventilation–perfusion (V–P) scans [2]. Tourassi et al. analyzed the Prospective Investigation of Pulmonary Embolism Diagnosis (PIOPED) database by testing ANN trained with three different techniques: cross validation, round robin, and bootstrap. The ANN was used do evaluate the diagnostic performances of artificial neural networks. The input space of the ANN consisted of 21 features extracted from clinical images by the physicians’ interpretations of V–P lung scans and chest radiographs (CXR). The study of the three Receiver Operating Characteristics curves (ROC) [3] did not show significant statistical differences among the techniques. The authors suggested the use of all three techniques for supporting the final diagnosis performed by physicians [4]. Digitized ventilation–perfusion scans correlated with CXR have been largely studied to check if a patient is affected or not by PE. Serpen et al., addressed the problem of diagnosing pulmonary embolism by developing a software for the automatic reconstruction of lung images from ventilation–perfusion scans. The software is based on Principal Component Analysis and Artificial Neural Networks. They noted that both techniques suffer varying degrees of performance degradation in high probability pulmonary embolism cases [5]. Other medical images, like the ones obtained from ventilation–perfusion (V–P) lung scintigrams have been used in the diagnosis of pulmonary embolism. Holst et al., proposed an artificial neural network, trained by 18 features extracted from a large set of V–P scintigrams. The performance of their network, expressed in terms of area under curve of the ROC, encouraged the use of ANN for PE diagnosis [6]. The performance of this ROC has been improved in a subsequent work, in which the authors trained three ANNs: the first network was trained using features obtained from each set of perfusion scintigrams, the second network was trained using features from each set of (joint) ventilation and perfusion studies in six projections, and the third network was trained using features from the perfusion study in six projections combined with a single ventilation image from the posterior view [7]. For a comparative analysis of the several models adopted for predicting the presence of acute pulmonary embolism a reading of [8, 9] is suggested. Beside these works that are completely based on ANNs trained with clinical images, an innovative approach has been proposed by Rucco et al. [10] in which the authors combined the topological reduction techniques for selecting the clinical variables that are used as input space for artificial neural network. In all these papers the performance of the classifiers are good enough to suggest that a Computer Aided Detection (CAD) for PE is reasonable, however the results of the Rucco et al. [10] paper can not be directly compared with the result of the two others because they used a different cohorts. In this work a new CAD is proposed based on the mathematical theory of Hypernetworks [11] and *Q-*analysis [11–14]. *Q*-analysis allows for a dimensional description of the patients dataset and then its results are used for feature selection to use in the training step of an artificial neural network. The dataset used in this work is the same used by Rucco et al. [10]. This study uses data from 28 diagnostic features of 1427 people considered to be at risk of having PE and the outcome for each person of whether or not they developed a PE. This approach correctly recognized 94 % of those developing a PE.

### Hypernetwork

Hypernetwork theory [11, 12, 15–17] concerns the formation of combinations of entities under relationships to form hypersimplices. For example, the combination of attributes $\langle elderly, poor sight, live alone\rangle$ may make a person more predisposed to a fall than, say, $\langle young, normal sight, cohabit\rangle.$ The entities in the brackets are called vertices since hypersimplices generalise networks. Two vertices $\langle a, b\rangle$ correspond to the usual edge in a network, three vertices correspond to a triangle $\langle a, b, c\rangle,$ four vertices correspond to a tetrahedron $\langle a, b, c, d\rangle,$ and so on. From this perspective it is natural to see the 28 features defined in Table 1 as a hypersimplex with 28 vertices, $\langle f_{1}, f_{2}, \ldots, f_{28}; R \rangle.$ The symbol R is the relation that binds the vertices together. Hypernetwork theory makes a distinction between a vertex with a measurement on it, and a vertex as a class of values associated with a scale. This also provides a coherent way of handling missing data. In the simplest case an observed variable may just be a categorical variable stating that a condition is either present or not-present. Rather than treat this as a single vertex with a number on it, 0 meaning not-present and 1 meaning present, hypernetwork theory treats this situation by having two vertices, ‘x is not present’ and ‘x is present’. Then for the case of missing data neither of the vertices is related to the patient. Note that ‘observed as not being present’ is very different from ‘not being observed’, and this affects subsequent analyses. In other cases the interpretation of data as a number associated with a vertex on a ratio scale may not reflect the way the data were collected and what they mean. Very often the numbers on the scale are ordinal and should be interpreted, for example, as low, medium and high classes defined by three vertices in this case: $x_{low}$, $x_{medium}$, and $x_{high}$. This is effectively a discretization of the scale. In hypernetwork terms this makes very good sense. Consider four pairs of diagnostic vertices $a, \sim a, b, \sim b, c, \sim c, d, \sim d$, where $\sim x$ means ‘not *x*’. In hypernetwork theory $\sim x$ is called the antivertex of *x*. When all the data are available every subject will be represented as a tetrahedron such as $\langle a, b, \sim c, d\rangle.$ Suppose that the data for *d* or $\sim d$ were recorded as *NaN*, with no information for either vertex. Then the simplex as $\langle a, b, \sim c\rangle$ represents that part of diagnostic space for which there is information. The simplex $\langle a, b, \sim c\rangle,$ which is a triangle and a face of the tetrahedra $\langle a, b, \sim c, d\rangle$ and $\langle a, b, \sim c, \sim d\rangle,$ is the best representation of the available data.

**Table 1:** The original 28 variables

1	ID	Patient’s identifier
2	Age	With the increase of the age, increase the incidence
3	N_F_Pred	Number of predictive factors
4	N_F_Risk	Number of risk factors
5	Previous DVT	A previous DVT / PE is a risk factor repeated infringement DVT / PE
6	Palpitations	Aspecific symptom. If it implies a tachycardia could be associated with DVT/PE
7	Cough	Coughs symptom very nonspecific but frequently present in patients with DVT / PE
8	dDimer	A value of d-Dimer <230ng = ml is associated with a low risk / absent of DVT/ PE. A very high value is associated with a high risk of DVT / PE
9	PAS	A low PAS is present in patients with DVT / PE and hemodynamic shock
10	PAD	In cardiogenic shock with DVT / PE is low, sometimes undetectable. By itself has no value despite of the PAS
11	FC	In the patient with TVP / EP tachycardia is often found
12	PAPS	It is one of the criteria of right ventricular dysfunction. It can be normal in the case of EP low entity.
13	WBC	The value increases with inflammatory forms (pneumonia, etc...) that can be confused with DVT / PE
14	Cancer at diagnosis	Cancer at diagnosis It is a risk factor for DVT / EP recognized
15	Troponin	It is a marker of myocardial infarction or heart failure and can be confused with DVT / PE
16	Shockindex	It is the ratio between PAS and FC, if it is greater than 1 is indicative of shock
17	NEOPLASIA	It is a risk factor for DVT / EP recognized
18	RVD	Right ventricular overload in the course of DVT / PE
19	Wells score	Wells score
20	Revised Geneva score	Revised Geneva
21	Wicki score	Wicki’ score
22	Dyspnea	Main symptom in DVT / PE
23	Chest pain	Chest pain is present in myocardial infarction, in pleural effusion, in the high DVT
24	PCO2	Associated with low pO2 may be suggestive of DVT / PE
25	PO2	Associated with low pCO2 may be suggestive of DVT / PE
26	PH	In DVT / EP pH is usually normal
27	Hemoptysis	It is the expectoration (coughing up) of blood or of blood-stained sputum from the bronchi, larynx, trachea, or lungs
28	Final diagnose	Final physicians’ diagnosis

### Artificial neural network

An artificial neural network is a computational metaphor inspired by the biological brain’s network. The neural network used in this study has a three-layer, feed-forward architecture and was trained by using the back-propagation algorithm with a sigmoid activation function. According to this learning scheme, the network tries to adjust its weights so that for every training input it can produce the desired output. A supervised learning strategy was used where during the training phase, the network is presented with pairs of input-output patterns. It has been shown that this technique minimizes the mean squared error (MSE) between the desired and the actual network output following an iterative gradient search technique. The number of hidden layer neurons was determined experimentally, evaluating each case by trial and error, as there is no theory to predict the number of neurones for the best topology of the network that ensures the optimal sensitivity and specificity [4, 18].

### The pulmonary embolism case study

PE is still difficult to diagnose because clinical symptoms and signs are nonspecific [1]. Among the patients who die of PE, the majority of the deaths are observed in the initial hours after the acute event [19]. Despite recent diagnostic advances, delays in pulmonary embolism diagnosis are common and represent an important issue [20]. As a cause of sudden death, massive PE is second only to arrhythmic death. Among survivors, recurrent embolism and death can be prevented with prompt diagnosis and therapy. Unfortunately, diagnosis is often missed because patients might show nonspecific signs and symptoms. If left untreated, approximately one third of patients who survive an initial pulmonary embolism die from a subsequent embolic episode [21]. Independently of the anatomic extension of the embolism, the vascular involvement and the dimensions of the thrombus, it is now widely accepted that the most important prognostic marker is hemodynamic compromise. Classically, PE has been subdivided in massive, hemodynamically unstable (shock, hypotension or cardiac arrest), sub-massive (normotensive with right ventricle dysfunction) or non-massive (normal blood pressure and no signs of right ventricle dysfunction). The highest mortality is observed in the first two categories. Shock, right ventricle dilatation at echocardiography and myocardial damage as assessed by Troponin I levels are deemed to be the most important and widely recognized elements that can predict adverse outcomes in PE. The role of “newer” markers, such as B-type natriuretic peptide (BNP), even if included in some PE prognostic models [22] and associated to a worse prognosis, is less clear. A significative increase of BNP levels can be interpreted as a global expression of a right ventricle dysfunction (RVD) [23]. These markers maintain a predictive role also among patients showing normal blood pressure and wiothout signs of shock: RVD, defined as the presence of increased troponin values or echocardiographic evidence of right ventricle dilatation or increased pulmonary pressure, has been associated with higher mortality [24] in all patient subsets. All the automatic methods proposed in literature [4–7] for PE diagnosis are in line with the guidelines [1] in proposing the use of imaging analysis, e.g., CT-angiography, as input space. The risk associated to CT-angiography is still under discussion [25], and its associated risks can not be neglected. Another drawback is the high cost of CT-angiography [26]. These reasons suggest that alternative methods for diagnosing PE without using imaging analysis are important and an opportunity for research. This work presents an approach that does not require imaging analysis and is based on CVs collected by other simpler means.

## Results and discussions

*Q-*analysis performs a comprehensive dimensional description of the dataset in terms of the existing relations between patients and clinical variables. This allows the identification of the structural properties (the backcloth) of the dataset in terms of the dimensionality of the connectivity of the clinical variables as function of the number of patients that share those same clinical variables [11, 27]. Naturally it is important to describe the dataset in terms of what happens to the clinical variable representing the final diagnosis of the patients (*Final diagnosis [0]* for negative diagnosis and *Final diagnosis [1]* for positive diagnosis).

**Fig. 1:**
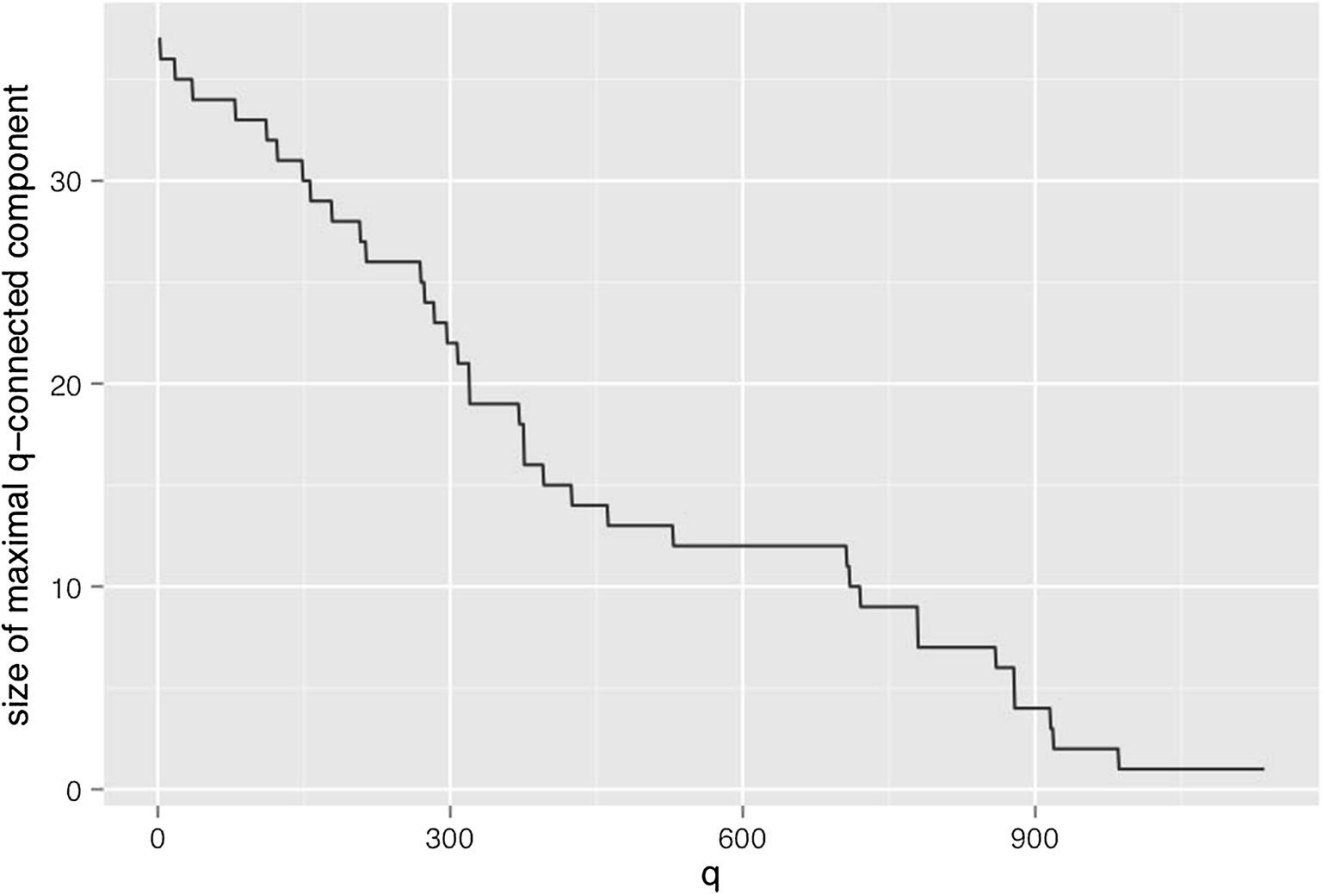
Size of maximal connected component as a function of the q-dimension

The positive diagnosis *Final diagnosis [1]* variable occurs 819 times while the *Final diagnosis [0]* occurs only 608 times. This means that they can only be *q-*connected up to dimensions of 818 and 607 respectively. It is therefore interesting to identify what happens to these two variables. Both are present in the same *q-*connected component up to the dimension $q=529$, when *Final diagnosis [0]* becomes an isolated variable and *Final diagnosis [1]* persists in a connected component with another 11 variables. This 12 element component persists until *Final diagnosis [0]* disappears at $q>607$ and beyond until $q=707$, where the component starts to slowly get disconnected. At $q=720$*Final diagnosis [1]* is still connected to 9 variables and it becomes isolated by $q=721$. It stays in isolated from that dimension until $q=817$ when it disappears (see Fig. 1).

The identification of the stability zone can be clearly observed in the Fig. 1 where the maximal size connected component is plotted as a function of the dimension of the induced *q*-graph.

The 12 factors identified in plateau in the *q-*range $=\,[529,707]$ the *Final diagnosis [1]* connected component are *age [1]*, *cough [0]*, *shockindex [0]*,* PO2 [1]*, *previous DVT [0]*, *palpitations [0]*, *dDimer [1]*, *FC [1]*, *dyspnea [1]*, *chest pain [0]*, *hemoptysis [0]*, and naturally *final diagnosis [1]*.

This backcloth reveals the connectivity of the variables at this dimension, but it is not an account of the causal or even correlation effect of those variables with the diagnosis. To infer these we used a neural network approach using as input all the existing variables.

In any case, *Q-*analysis reveals that this high dimensional component of the variables is a subset that is shared among many patients making them general and non-discriminative in terms of diagnose, for short we call this set “connected higher dimensional set”.

In order to automatize the diagnosis of PE, in Matlab [28] three ANNs were trained with three different input spaces. For each network a common strategy was followed to find the optimal architecture: 76 combinations of neurons were tested in the hidden layer, from 1 neuron to 77, and for all these we computed the k-fold cross validation (with k = 10) between the training and the testing sets [29]. From the entire dataset of patients 85 % were selected, with the attributes used in the *Q-*analysis, this subset is divided in two sets (training and testing) by the *k-*fold procedure. It is important to remark that in order to avoid redundant information, in all the three cases the scores already computed by MDs (Wells, Revised Geneva and Wicki) are not used. Once the optimal configuration (number of neurons in the hidden layer, weight matrix) is found, it is applied to the remaining 15 % (the validation). The output of the classifier over the validation test is compared with the final diagnosis assessed by clinicians via Jaccard coefficient (Eq. 1) [30], that is basically the ratio between the cardinality of the intersection between the classification set of the medical doctor and the output of our classifier, and the number of elements belonging to the union between the responses of the medical doctor with the output of our classifier.

1 $$\begin{aligned} J(Ann,MD)=\frac{|Ann \cap MD|}{|Ann \cup MD|} \end{aligned}$$

where *MD* is the classification assessed by MDs and *Ann* is the automatic classification.

**Fig. 2:**
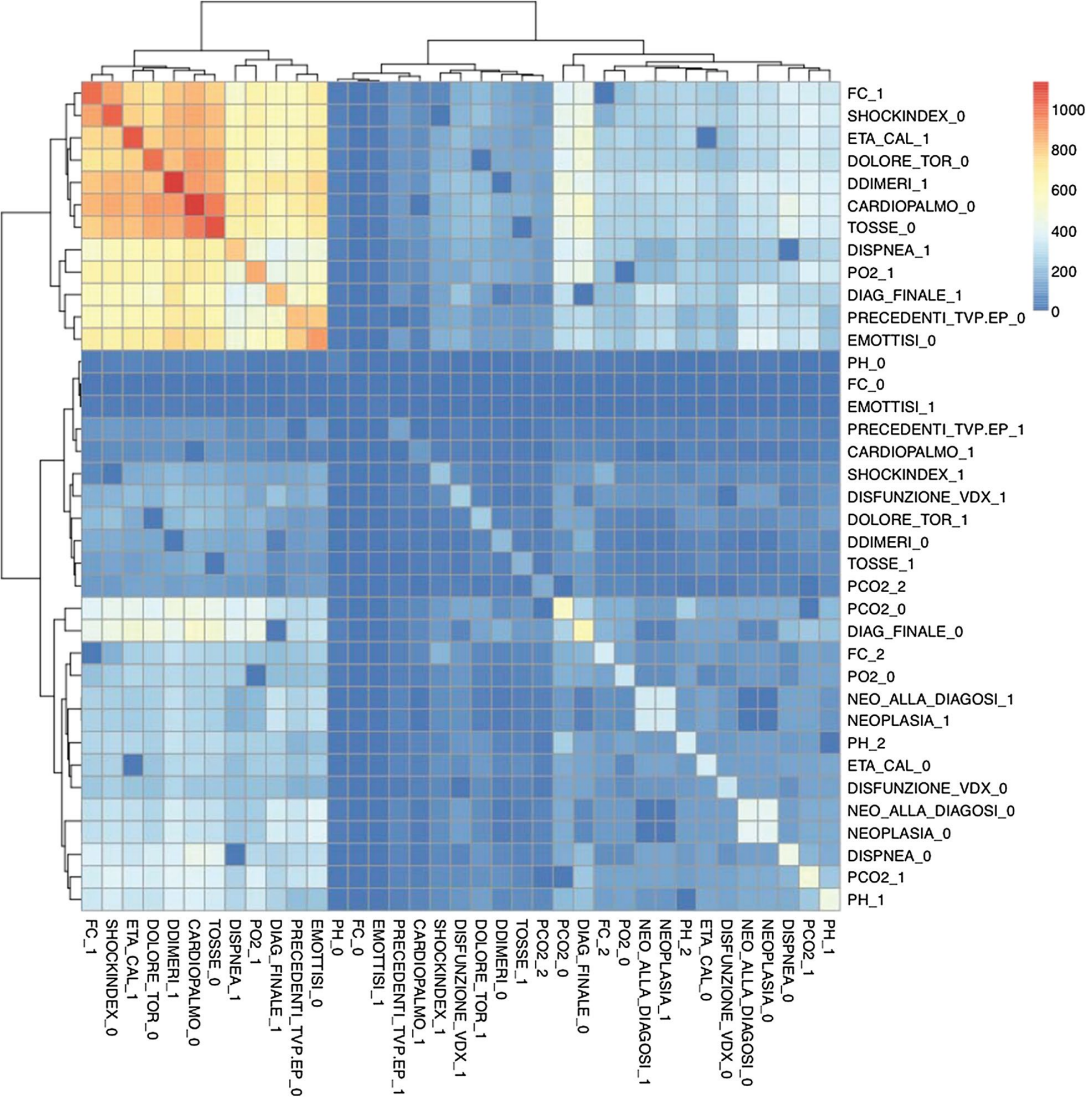
Clustering of the shared face matrix of the pulmonary embolism variables

**Fig. 3:**
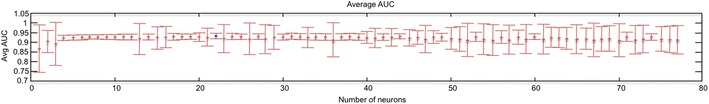
Study of number of neurons: y-axis average AUCs, x-axis number of neurons. The *blue point* represents the optimal configuration

**Fig. 4:**
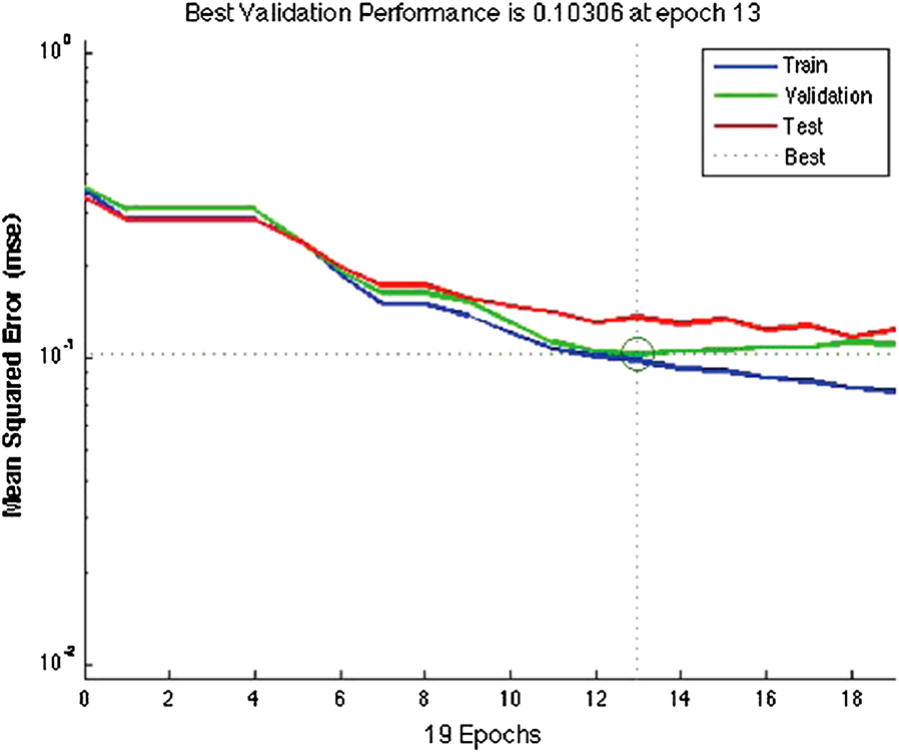
Best mean squared error (MSE) at 13 epochs

**Fig. 5:**
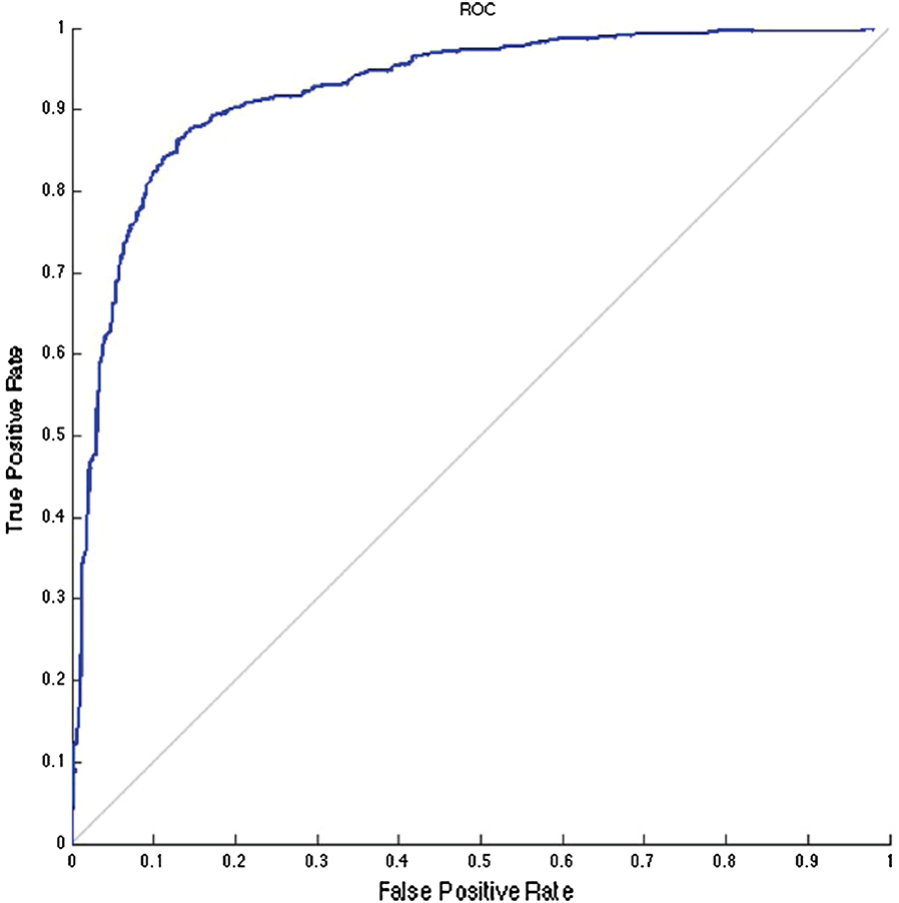
ROC curve for the new classifier based on Artificial neural network with AUC = 93 %

1. An ANN was trained with the features belonging to the “connected higher dimensional set”, the AUC of the corresponding ROC is 60 % rate = 0.0453, Jaccard = 55 %. In this case the network configuration was: 12 input neurons, five neurons in the hidden layer, oneneuron in the output layer.
2. The variables that are not connected (unconnected higher dimensional level set) in an higher dimensional level are therefore very important for inclusion in the ANN training. In this case the ANN was equipped with 17 input neurons, eight neurons in the hidden layer, one neuron in the output layer. The performance of the ANN trained with the complementary set of variables yielded an AUC of 86 % rate = 0.0200, Jaccard = 72 %. This confirms that the diagnose can not be made by the structural backcloth of the relation between the variables, but by the traffic existing in the structure (see Fig. 2). This result is clearly observed also by clustering the elements of the shared face matrix with an Euclidean distance as in Fig. 2. It is clear from the clustering that the top-left block is highly correlated and share the highest number of patients.
3. An artificial neural network was trained using the full thresholded dataset that is the union of “connected higher dimensional set with unconnected higher dimensional set”. This network is equipped with 22 neurons in the hidden layer (Fig. 3), and an output layer with a single decision node. The learning rate was selected to be 0.5 and the momentum coefficient to be 0.9, the optimal number of iterations (epochs) have been found equal to 13. It corresponds to a Mean Squared Error (MSE) equal to 0.10306 (see Fig. 4). The AUC of the ROC curve (see Fig. 5) is equal to 93 % rate = 0.0564, Jaccard = 89 %.

**Fig. 6:**
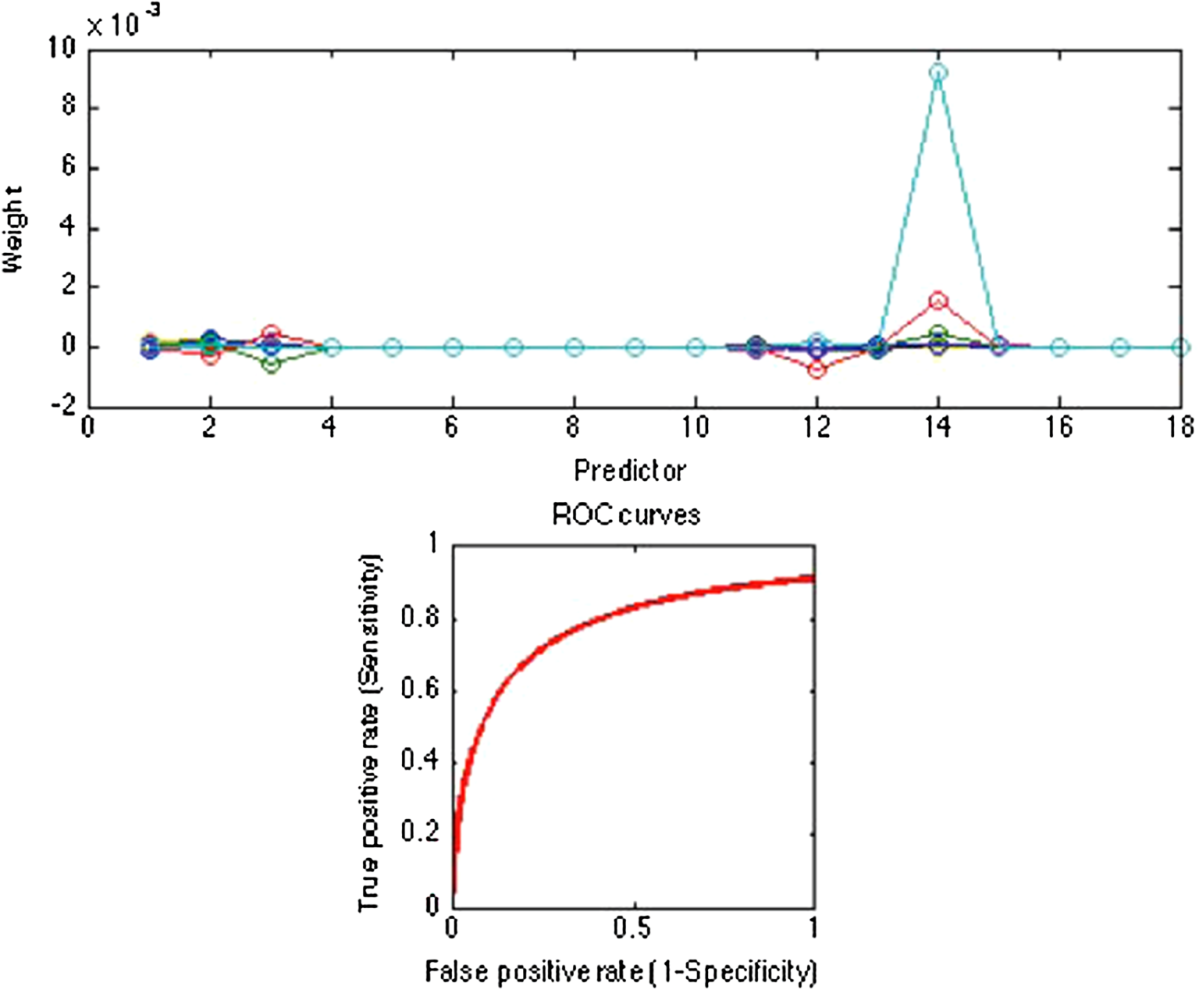
Weight contributes in each component. *Bottom* ROC curves for a PLS with 18 components

In this study, the network’s output is interpreted as the probability of PE being present. The results can be compared to the findings of previous works that have taken different approaches. The comparison is summarized in Table 2 and clearly shows that this novel approach supplants previous results. In detail we studied the performance of the two scores: revised Geneva and Wells. The scores have been used independently to train an artificial neural network. An alternative approach for the features selection has been performed. We used the Mood’s variance test and the relative* p* value to highlight the feature set with more discriminating power between the healthy and pathological classes. The authors have found results completely comparable with the literature (i.e. d-Dimer is the clinical variable that must be used as first clinical test in the pulmonary embolism diagnose procedure). With these extracted features an ANN was trained with the selected features [31]. A Partial Least Square Regression [32] was tested and the best performance has been obtained using the entire set of components (18) due to the low variance expressed by each single variable. The number of components has been found iteratively: for each iteration the rows of the initial dataset have been shuffled randomly and both the Jaccard coefficient (between the fitted response and the expected response) and the AUC have been evaluated (see Fig. 6).

**Table 2:** Comparison of performance

	Wells + ANN	Rev. geneva + ANN	Stat. feature reduction + ANN	Conn. higher dimen. set + ANN	PLS regression	Unconn. higher dimen. set + ANN	Ayasdi iris + ANN	Neural hypernetwork
AUC (%)	0.74	0.55	0.45	0.6	0.74	0.86	0.89	0.93

The occurrence of Pulmonary Embolism in the population dataset seems to be higher than in other works [33, 34] in which the mean onset is between 10 and 30 %. The reason for this is that the dataset is constructed from the patients arriving in the department of Internal and Subintensive Medicine after a first screening executed by the emergency room team. The emergency room team claimed that all these patients were affected by PE, while the subsequent analysis with CT-angiography showed that only the 58 % were really affected by PE. The dataset includes all patients that were sent to the department from the emergency room team.

## Conclusions

This paper introduces a novel integrative approach for the analysis of partial and incomplete datasets based on *Q*-analysis simplicies’ descriptors in terms of the *augmented vertex set* (vertices $\cup$ antivertices) that is coupled with machine learning [11, pp. 69–70]. The new approach, called Neural Hypernetwork, has been applied to a case study of pulmonary embolism diagnosis with great success as shown by the 93 % of the AUC of the ROC (Fig. 5) on the testing set and a Jaccard coefficient equal to 89 % between the MDs final decision and the automatic classification on a validating set. To improve the statistics reliability of this work it is planned to repeat the study using cohorts coming from different Hospitals. The purpose of this study was to introduce an innovative hybrid approach based on the hypernetworks’ theory and supervised artificial neural networks. This method has been used to design a new CAD for pulmonary embolism with the aim to reduce the number of CT-angiography analysis and ensuring a good efficiency of the diagnose. It is important to underline that the results obtained in this study are affected by bias due to the population of the analyzed patients. The patients forming the database were selected by the MDs of the emergency room as patients with high risk of PE. Fortunately only a subsample exhibited the pathology.

Future investigation will be focusing on the use of the *S*[*B*]-paradigm for characterizing the Cases 1 and 2 described in "Results and discussions" Section. The *S*[*B*] paradigm [35, 36], is a computational approach for modeling complex systems. The paradigm is based on two interacting levels: *Structural* and *Behavioral*. The two levels are entangled by a feedback loop: *S* contains a set of constraints for the observables, the observables set (at *B* level) can work regarding the constraints or evolve highlighting the emergence of new models. Within the *S*[*B*] it is easy to imagine the *artificial neural network* as the structural level, namely the global description of the system based on the interaction among all the clinical variables, and the *hypernetworks* with their *Q-analysis* synthesis as the behavioral level.

## Methodology

### Ethics declaration

The study was approved by the ethics committee of the Marche Polytechnic University. All participants or caregivers gave their informed written consent according to the Declaration of Helsinki.

### Missing data and discretization in machine learning

Discretization of continuous features plays an important role in machine learning techniques either because the machine learning technique itself requires a nominal feature space or because discretization allows better results of the operating machine learning technique. The field of research on dataset discretization for machine learning is vast and beyond the scope of this paper, but it is important to say that such algorithms usually aim to maximize the interdependency between discrete attribute values and class labels, as this minimizes the information loss due to the discretization process. The process of discretization has to balance the trade off between these two goals and many studies have shown that several machine-learning techniques benefit from this discretization process [37–39]. In the present study, the transformation process of the dataset by discretization and duplication of the features variables (a process drawn from *Q*-analysis *augmented vertex set*) aims also to improve quality of the machine learning technique in dealing with missing data.

In the present study, the original dataset is very incomplete with 24 % of missing data. This forces the use of some data transformation technique that allows the use of the entire dataset with machine learning techniques. Dealing with missing data presents a challenge and many techniques can be used to overcome these difficulties. Typically missing data can be categorized according to the statistical properties of the occurrences of the missing data. Either the missing data is missing completely at random (MCAR, independent of that variable y or other variables x), is missing at random (MAR, missing data depends on other variables x), or it is missing not at random (MNAR, dependence of self variable and others). Several techniques can be applied to deal with the different types of missing data and the most common are deletion methods, imputation methods and model based methods. Deletion methods remove the missing data from the dataset either in a listwise manner or a pairwise manner. This kind of procedure introduces a bias in the resulting dataset if the missing data is of type MNAR. Imputation methods generate values for missing data from statistical measures like sample mean and mode and are therefore difficult to apply to class variables. Also they reduce variability and weaken covariance and correlation estimates in the data because they ignore relationships between variables. Model-based methods usually use maximum likelihood estimators or multiple imputations. Although dealing with the entire dataset, the estimated dataset present problems of bias in the case of MNAR. Taking this into consideration we developed a mechanism of dealing with the missing data in the dataset. The dataset is naturally MNAR as the missing data is highly dependent on the observed variable as it is also dependent on other variables. This means that deletion methods will reduce (drastically) the number of observations and will introduce bias. The imputation and model-based methods are also of difficult application due to the existence of nominal categories in the dataset. By the combination of discretization (needed to improve machine learning techniques) we were able to solve also the problem of the missing data in the dataset.

The discretization was done manually dependent on medical indications of what would consist health risk for each factor studied. For each factor a pair of boolean variables were created that represent if the observed factor “does not contribute to an health risk” and if the observed factor “contributes to an health risk”. In terms of *Q*-analysis, this corresponds to an explicit transformation of each variable into a vertex, and its antivertex generating what is called the *augmented vertex set* [11, pp. 69–70]. This technique allows information to be kept about missing data by setting missing data entries into those pairs of vertices as $\langle false,false\rangle$ ($\langle 0,0\rangle$ for computation purposes). On the other hand if there is an observation for that factor it will be represented in these pairs of vertices $\langle false,true\rangle$ or $\langle true,false\rangle$($\langle 0,1\rangle$ or $\langle 1,0 \rangle$ for computational purposes). These factors are discretized by taking into account the risk ranges discussed with the medical staff and therefore represent a data driven solution for the discretization problem. In this way one does not incur in the problems of traditional discretization techniques. The resulting dataset includes more information about the original dataset then a deletion technique would produce and therefore the subsequent machine learning technique benefits from the maximum number of samples in the dataset used during the learning phase of the algorithm.

### Dataset description

The dataset of the pulmonary embolism is constituted by 1430 samples corresponding each entry to a patient and 28 variables, where 26 of these variables are clinical indicators. One variable corresponds to the patient ID and one variable indicates the final disease diagnostic on the pulmonary embolism condition (see Table 1).

The original dataset includes approximately 24 % of empty elements (NaN). These are elements for which a value is unknown either because the patient was not asked or because they did not provide an answer. The distribution of the missing data is not uniform and in the case of some variables they represent 1227 out of the 1430 observations (86 %). A detailed breakdown of how the different variables are affected is found in Table 3. From the original dataset a subset of 19 clinical variables has been selected. The following were discarded:

- ID has been used only to identify uniquely each patient;
- Troponin, PAS, PAD and PAPS because they represent information already deduced from other clinical variables or due to the huge amount of missing data (PAPS);
- Wells, Revised Geneva and Wiki score because they have been thought as the synthesis of other clinical variables plus the MDs opinion and they represent the clinical prediction rules actually used in the hospital.
- Final diagnosis has been used during for labeling the input for the artificial neural network

**Table 3:** NaNs percentage

Feature	NaNs	% of NaNs
ID	0	0.00
Age_CAL	1	0.07
N_F_PRED	95	6.64
N_F_Risk	108	7.55
Previous DVT	490	34.27
Palpitations	196	13.71
Cough	184	12.87
dDimer	111	7.76
PAS	162	11.33
PAD	546	38.18
FC	58	4.06
PAPS	1227	85.80
WBC	38	2.66
Cancer at diagnosis	670	46.85
Troponin	571	39.93
Shockindex	170	11.89
Cancer	668	46.71
RVD	862	60.28
Wells score	512	35.80
Revised Geneva score	0	0.00
Wicki score	665	46.50
Dyspnea	186	13.01
Chest pain	184	12.87
PCO2	242	16.92
PO2	238	16.64
PH	577	40.35
Hemoptysis	490	34.27
Final diagnosis	0	0.00
Total	9251	23.10

The application of the thresholding procedure projected the 19 clinical variables in 38 descriptors (see Table 4).

**Table 4:** Thresholding of the clinical descriptors

1. Age[0] = $\{Age |Age \le 64 \}$	2. Age [1] = $\{Age | Age \ge 65 \}$
3. N_F_Pred[0] = $\{N\_F\_Pred |N\_F\_Pred =0 \}$	4. N_F_Pred[1] = $\{N\_F\_Pred | N\_F\_Pred \ge 1 \}$
5. N_F_Risk[0] = $\{ N\_F\_Risk| N\_F\_Risk =0 \}$	6. N_F_Risk[1] = $\{ N\_F\_Risk| N\_F\_Risk \ge 1 \}$
7. Previous DVT[0] = $\{Previous\,DVT| Previous\,DVT =0 \}$	8. Previous DVT[1] = $\{Previous\,DVT| Previous\,DVT =1 \}$
9. Palpitations[0] = $\{Palpitations| Palpitations =0 \}$	10. Palpitations[1] = $\{Palpitations| Palpitations =1 \}$
11. Cough[0] = $\{Cough| Cough =0 \}$	12. Cough[1] = $\{Cough| Cough =1 \}$
13. dDimer[0] = $\{dDimer| dDimer \le 230 \}$	14. dDimer[1] = $\{dDimer| dDimer > 230 \}$
15. FC[0] = $\{FC|50 \le FC \le 99\}$	16. FC[1] = $\{FC| FC \ge 100\}$
17. WBC[0] = $\{WBC|2000 \le WBC \le 10000\}$	18. WBC[1] = $\{WBC|WBC \ge 10000\}$
19. Cancer at diagnosis[0] = $\{ Cancer\,at\,diag.| Cancer\,at\,diag. =0 \}$	20. Cancer at diagnosis[1] = $\{ Cancer\,at\,diag.| Cancer\,at\,diag. =1 \}$
21. Shockindex[0] = $\{Shockindex|0.0 \le Shockindex\le 0.89\}$	22. Shockindex [1 ] = $\{Shockindex| Shockindex\ge 0.9\}$
23. Cancer[0] = $\{Cancer| Cancer=0 \}$	24. Cancer[1] = $\{Cancer| Cancer=1 \}$
25. RVD[0] = $\{RVD| RVD=0 \}$	26. RVD[1] = $\{RVD| RVD=1 \}$
27. Dyspnea[0] = $\{Dyspnea| Dyspnea=0 \}$	28. Dyspnea[1] = $\{Dyspnea| Dyspnea=1 \}$
29. Chest pain[0] = $\{Chest\,pain| Chest\,pain=0 \}$	30. Chest pain[1] = $\{Chest\,pain| Chest\,pain=1 \}$
31. PCO2[0] = $\{PCO2| 35 \le PCO2 \le 45 \}$	32. PCO2[1] = $\{PCO| PCO2 > 45 \}$
33. PO2[0] = $\{PO2| PO2 \le 60 \}$	34. PO2[1] = $\{PO2| PO2 > 60 \}$
35. PH[0] = $\{PH| 7.3 \le PH \le 7.42 \}$	36. PH[1] = $\{PH|PH > 7.42\}$
37. Hemoptysis[0] = $\{Hemoptysis| Hemoptysis=0 \}$	38. Hemoptysis[1] = $\{ Hemoptysis|Hemoptysis=1 \}$
39. Final diagnosis[0] = $\{Final\,diagnosis| Final\,diagnosis=0 \}$	40. Final diagnosis[1] = $\{Final\,diagnosis| Final\,diagnosis=1 \}$

## Abbreviations

PE: pulmonary embolism
MDs: medical doctors
CPR: clinical prediction rules
CV: clinical variables
CAD: computer aided detection
PIOPED: prospective investigation of pulmonary embolism
RVD: right ventricular disease
MSE: mean squared error
ANN: artificial neural network
ROC: receiving operating curve characteristics
